# Recovery of haemal lordosis in Gilthead seabream (*Sparus aurata* L.)

**DOI:** 10.1038/s41598-019-46334-1

**Published:** 2019-07-08

**Authors:** Stefanos Fragkoulis, Alice Printzi, George Geladakis, Nikos Katribouzas, George Koumoundouros

**Affiliations:** 1 Biology Department, University of Crete, Herakleion, Crete, Greece; 2 Andromeda S.A., PEO Patron-Athinon 55, Agios Vasilios, 26500 Rion, Greece

**Keywords:** Ichthyology, Bone remodelling

## Abstract

Haemal lordosis is a frequent abnormality of the vertebral column. It has been recorded to develop in different finfish species, during the hatchery rearing phase. Under certain conditions, this abnormality reaches a high prevalence and severity degree, with significant effects on the external morphology of the fish. We show that haemal lordosis recovers during the on-growing of Gilthead seabream in sea cages. At the end of the hatchery phase, 1700 seabream juveniles were tagged electronically and examined for the presence of haemal lordosis. Subsequently, their morphology was examined periodically up to the end of the on-growing period. We found that the prevalence of fish with a lordotic external morphology decreased during the studied period by approximately 50%. Interestingly, 27% of the recovered fish presented a completely normal vertebral column. Geometric morphometric analysis showed no significant differences in the body shape between the fish with a recovered normal phenotype and the fish that were normal since the beginning of the on-growing period. Our results provide the first evidence for the recovery of lordosis during the growth of fish. A mechanism with multiple levels of remodeling of abnormal bones is suggested.

## Introduction

The presence of skeletal abnormalities in reared fish is a major problem of product quality, with significant effects on fish morphology and biological performance^1^. Although early sort-out of the abnormal juveniles minimizes the negative effects of abnormalities on the quality of the final product, this leads to a significant financial loss for hatcheries (e.g. over 50 million € per year for the Mediterranean aquaculture^1^). In most of the species, skeletal abnormalities develop during the embryonic, larval and early juvenile stages^2–6^, due to unfavorable rearing conditions^7–11^, genetic factors^12–14^ and/or due to occasional accidental deviations from the standard operating procedures. Existing literature suggests that prevalence of skeletal abnormalities might be decreased with the growth of the fish, as a result of the high lethality of some deformity types (e.g. prehaemal kyphosis^15^; haemal vertebral compression and fusion^3^). Recovery of skeletal abnormalities has only been recorded for the case of gill-cover abnormalities of light severity in Gilthead seabream^16^ and Atlantic salmon^17^. To our knowledge, there is no evidence on the recovery of vertebral abnormalities in finfish.

Vertebral lordosis (V-shape curvature) is a frequent abnormality of the reared finfish. Depending on the abnormality severity, the effects of lordosis on the external morphology display a continuous distribution, ranging from insignificant to severe body-shape alterations^18^. In physoclistous fish, lordosis was initially shown to affect the prehaemal vertebrae and arise from the failure of swimbladder inflation^19,20^. As it was later reported in *Dicentrarchus labrax*^21^ and *Pagrus major*^22^, lordosis may also develop in fish with a normally inflated swimbladder, on the haemal part of the vertebral column, as a result of high swimming activity. Nowadays, prehaemal lordosis is considered as a solved problem for finfish hatcheries, through mainly the application of appropriate methodology for the successful inflation of the swimbladder^1^. Concerning haemal lordosis, existing literature suggests that controlling the swimming intensity of the early juveniles might not completely solve the problem, since more factors have been shown to be significantly involved in its development (e.g. developmental temperature^10,23^; retinol levels in the larval diet^24^; genetic background^12^).

We examined whether haemal lordosis is further evolving during the growth of fish, in terms of its prevalence and effects on body shape. To reach this goal, we electronically tagged a juvenile population of Gilthead seabream (*Sparus aurata* L.) at the end of the hatchery phase and we followed fish morphology up to the end of the on-growing period. Selected fish is a major species of the European aquaculture, with a production of 161 thousand tons and 700 million juveniles in 2016 (source: FEAP).

## Materials and Methods

### Experimental design and fish origin

One thousand seven hundred seabream juveniles (86 ± 7 mm standard length, SL) were tagged electronically (FDX-B, Trovan Ltd, USA). Fish morphology was examined periodically until the end of the on-growing period (commercial size, 262 ± 14 mm SL) (Table 1). During each sampling, all specimens were anaesthetized by bath immersion (ethyleneglycol-monophenylether, Merck, 0.2–0.5 mL L^−1^), photographed on the left side and scanned for ID recognition before returning to the sea cage. All photographs were taken by means of a Canon PowerShot G9 camera, mounted on a tripod and positioned perpendicularly to the specimens. In each sampling period, missing fish were recorded. The significance of the differences in mortality rate between the two groups of fish (i.e. lordotic and normal) at each sampling age was tested by G-test^25^.

**Table 1:** Age, Standard Length (SL, mean ± SD) and number of fish which were photographed in each sampling period. Of the initially 1700 tagged fish, 1376 survived up to the end of the examined period.

Age (days post tag)	SL (mm)	°C days	n	Method of examination
1	86 ± 7	24	1700	Ext
77	142 ± 8	1934	1605	Ext
282	201 ± 13	5572	1507	Ext
371	240 ± 17	7209	1438	Ext
434	262 ± 14	8816	1376	Ext/x-rays

Ext, external morphology. °C days, the product of age with water temperature.

The examined group of fish originated from a common larval population and egg batch. Fish were reared according to the standard methodology followed by commercial hatcheries and cage farms for juvenile production and on-growing respectively^26^. At the end of the hatchery phase, 2000 juveniles (6.1 ± 1.9 g mean weight, 137 days post-hatching, dph) were transported to a commercial sea cage (6 m length, 6 m width, 8 m depth) for on-growing. Larvae were reared at an initial stocking density of 100 individuals per litre, in the presence of background phytoplankton (*Chlorella* sp.), with feeding on rotifers *Brachionus plicatilis* (5–27 dph), *Artemia* sp. nauplii (17–40 dph), and finally on inert commercial diets (>27 dph). Water temperature was maintained between 19–21 °C during the hatchery period, whereas during on-growing period it followed the natural seasonal fluctuations (Fig. S1). Rearing of Gilthead seabream embryos, larvae and juveniles was performed under routine production conditions at Andromeda S.A. This company is registered (registration number GGN 5200700699992) for aquaculture production in Greece and has secured a GLOBALG.A.P quality certification, which requires a certified Veterinary Doctor to periodically verify fish health and welfare. Animal sampling followed routine procedures and samples were collected by a qualified member of staff from a standard production cycle. The legislation and measures implemented by the commercial producer complied with existing Greek (PD 56/2013) and EU (Directive 63/2010) legislation (protection of animals kept for farming). Production and sampling, by an experienced worker, were optimised to avoid unnecessary pain, suffering or injury and to maximise larval survival.

### Categorization of the external morphology

**Figure 1:**
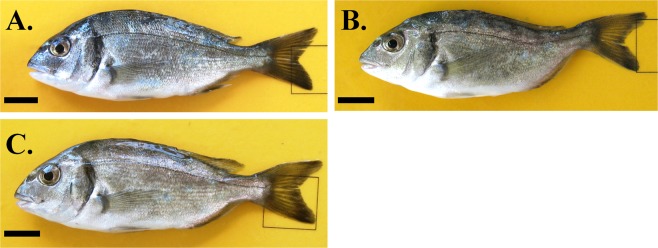
Representative cases of seabream juveniles, at 1 day post-tagging, with normal (**A**), lordotic (**B**) and uncertain (**C**) external morphology. Categorization was primarily based on the dorsal shift of the caudal peduncle in the abnormal fish (**B**). Scale bars equal to 1 cm.

Morphological examination of lordosis was carried out by three independent observers and it was blind in respect to fish pit-tag number. Discrimination of the fish with lordotic external morphology from those with a normal one was based on the dorsal shift of the caudal peduncle in the abnormal fish^23^ (Fig. 1A,B). Fish without a typical normal or lordotic external morphology were categorized as of uncertain phenotype (Fig. 1C).

To validate the methodology followed for the examination of external morphology, a random sample of 100 juveniles was taken from the source population, simultaneously to the tagging of experimental fish. Sampled juveniles were anaesthetized, photographed and stored at −20 °C. Based on their external morphology, juveniles were scored as normal, lordotic or of uncertain phenotype (Fig. 1). Subsequently, the results of phenotypic scoring were radiographically validated.

Based on the evolution of external morphology during the studied period, 146 adults were taken at 434 days post-tagging (dpt) and radiographically examined to further analyze and verify the observed external morphology. Radiographed adults were representative samples of fish with different external phenotypes (Table 2). All samples collected for x-raying, were euthanatized with an overdose of anesthetic. Radiography was performed with 50 KV voltage, 400 mA intensity and 0.002 s exposure time (Econet medical PpP-60HF, AGFA CR10).

**Table 2:** Number of adult fish which were radiographed at 434 days post-tagging.

Final external phenotype (434 dpt)	Initial external phenotype (1 dpt)	Total	Number of radiographed fish	% of fish radiographed
N	N	1199	25	2
	Un	25	13	52
	L	61	41	67
Un	N	3	—	—
	Un	4	2	50
	L	13	11	85
L	N	0	—	—
	Un	5	5	100
	L	66	49	74

A representative number of fish was randomly taken by each group, on the basis of the evolution of external morphology from tagging (1 day post-tagging) to the end of the trial (434 days post-tagging). N, fish with a normal external morphology. Un, fish with uncertain external morphology. L, fish with lordotic external morphology. dpt, days post-tagging.

### Morphometric analyses

Differences in body shape between juveniles (1 day post-tagging, dpt) with a normal, lordotic or uncertain external morphology were examined by geometric morphometry. Analysis included all the 174 juveniles of lordotic and uncertain external morphology (Table 2), together with a random sample of 150 juveniles with normal morphology. A similar analysis was performed on the 71 lordotic fish and the 17 fish of uncertain morphology at the final sampling (434 days post-tagging, Table 2), together with a random sample of 50 fish with normal morphology.

Differences in body shape between adults that recovered from lordosis and those with a normal external morphology since the beginning of the trial were examined by geometric morphometry. Analysis included all the x-rayed adults with recovered phenotype and 25 randomly selected and x-rayed normal adults (Table 2). All the x-rayed adults with a severe lordosis were also included in the study. Finally, an additional morphometric analysis was performed on the lordotic juveniles (1 day post-tagging), which were classified on those maintaining their external lordotic phenotype (sevL, severly lordotic) up to the end of the on-growing period, and juveniles that presented a recovered normal phenotype at 434 days post-tagging. The study also included 33 randomly selected normal juveniles.

**Figure 2:**
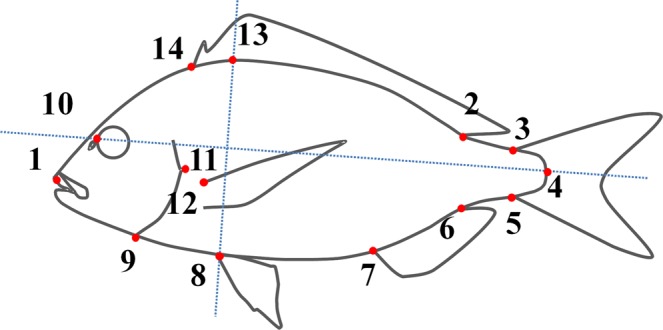
Landmarks collected in the present study. 1, Anterior tip of upper jaw; 2, posterior base of the dorsal fin; 3, dorsal tip of the base of caudal fin; 4, base of the central caudal lepidotrichium; 5, ventral tip of the base of caudal fin; 6, 7, posterior and anterior base of the anal fin, respectively; 8, base of the pelvic fins; 9, ventral tip of the gill cover; 10, anterior margin of the eye, dorsally to the nostril; 11, posterior tip of the gill cover; 12, dorsal base of the left pectoral fin; 13, projection of the landmark 8 on the dorsal profile of fish, perpendicularly to the axis which is defined by landmarks 4 and 10. 14, anterior base of the dorsal fin. (modified from Fragkoulis *et al*. 2017).

In all analyses, fourteen landmark measurements were taken on the digital photographs of the examined fish using tpsDig2 software^27^(Fig. 2). In order to adjust the individuals for centroid size and remove from the landmark configurations any effect irrelevant to shape, a generalized least square method was applied (coordGen6H software package^28^). TpsRelw software^29^ was used to calculate the weight matrix (partial warps, with uniform and non-uniform components of shape variation). Canonical variate analysis on the weight matrix was applied to test the effect of lordosis on body shape. Finally, to visualize the variation between the groups, spline diagrams were obtained after the regression of shape components on the canonical scores (tpsRegr software^30^).

## Results

### Validation of the phenotypic categorization by means of external morphology

**Figure 3:**
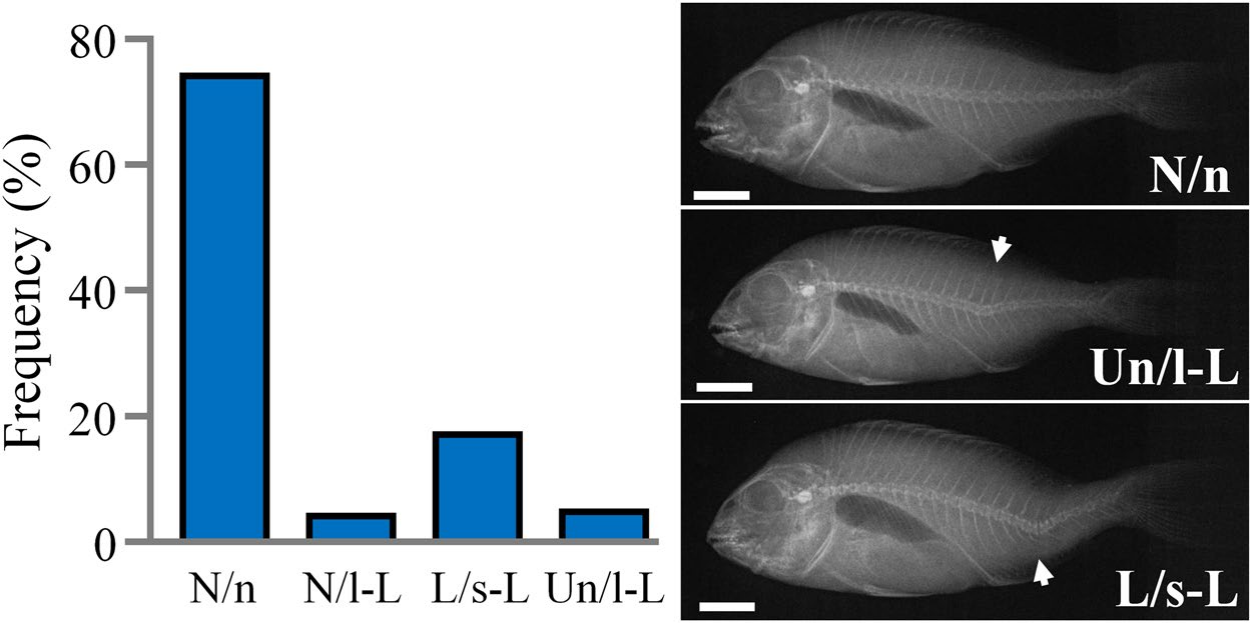
Frequency of the fish with normal (N), lordotic (L) and uncertain (Un) external morphology in the validation sample. Following the phenotypic categorization by means of external morphology, all individuals were examined in association with the radiographic appearance of the vertebral column (n, normal; l-L, lordosis of light severity; s-L, severe lordosis). Arrows indicated the lordosis center. Scale bars are equal to 1 cm.

Examination of the external morphology of the 100 juveniles sampled at 1 day post-tagging, revealed that 78 fish were normal (N) and 17 lordotic (L), whereas 5 fish presented an uncertain phenotype (Un) (Fig. 3). The following radiographic examination of the specimens showed that of the 78 N fish, the 74 had a completely normal vertebral column (N/n, Fig. 3), whereas the remaining 4 fish had a lordosis of light severity (N/l-L) (Fig. 3). All fish with lordotic external morphology (L group) presented a severely lordotic vertebral column (L/s-L, Fig. 3). Finally, the five specimens with uncertain external morphology, presented lordosis of light severity (Un/l-L, Fig. 3). In overall, external phenotypic scoring was verified by the radiographic examination in the 95% of normal and 100% of lordotic fish. The applied method of external phenotypic scoring failed to detect the four fish with light internal lordosis, which were categorized as of normal external phenotype (N/l-L).

### Evolution of haemal lordosis frequency during the on-growing period

**Figure 4:**
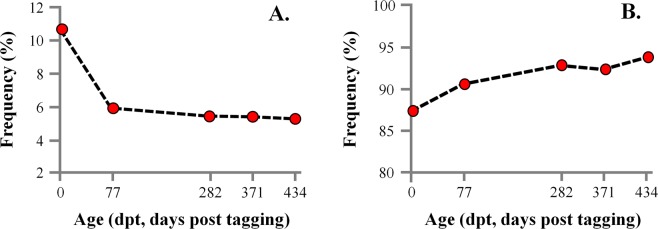
Frequency of the lordotic (**A**) and normal (**B**) fish in the different sampling periods. Categorization was performed on the basis of fish external morphology.

At tagging, phenotypic categorization of the 1700 fish by means of external morphology, showed that the 10.5% of them were lordotic and the 87.0% normal, whereas the 2.5% of the fish were categorized as of uncertain morphology. During the examined on-growing period, the frequency of lordotic fish substantially decreased to 5.8% at 77 dpt (days post-tagging) and 5.2% at 434 dpt (Fig. 4A). The frequency of normal fish increased to 90.2% at 77 dpt and 93.4% at 434 dpt (Fig. 4B).

**Figure 5:**
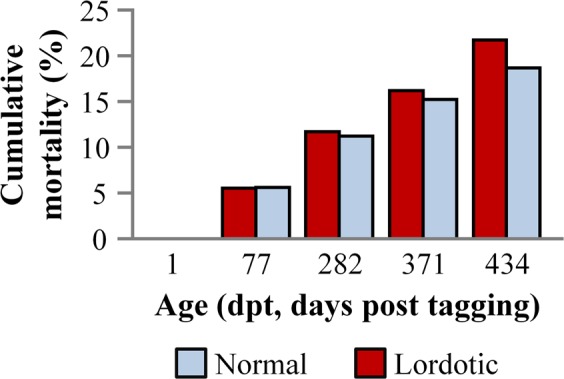
Cumulative mortality of normal and lordotic fish, as they were scored at tagging. No significant differences were observed in the mortality rate between the two groups of fish (p > 0.05, G-test).

Changes in the prevalence of normal and deformed fish could not be attributed to a comparatively higher mortality rate of the latter. Results demonstrated that no significant differences existed in the mortality rate between normal and lordotic fish (p > 0.05, G-test). Cumulative mortality rate was 18.7% for normal and 21.8% for lordotic fish (as they were phenotypically scored at tagging) (Fig. 5).

### Recovery of haemal lordosis during the on-growing period (1–434 dpt, days-post-tagging)

**Figure 6:**
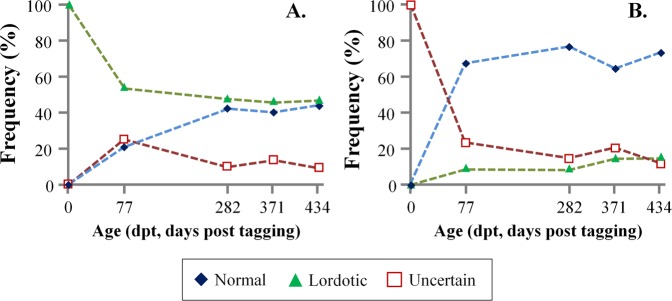
Evolution of the external phenotype of the initially abnormal juveniles (at tagging) throughout the on-growing period. (**A**) Fish with lordotic external morphology at 1 day post-tagging. (**B**) Fish with uncertain external morphology at 1 dpt. During the growth of the fish, the frequency of the lordotic and unclear fish was decreased in favor of the normal fish.

Monitoring of the external morphology of each abnormal specimen during fish growth, revealed that 20.7% of the fish with lordotic morphology turned into normal phenotype at 77 days post-tagging (dpt). By the end of the on-growing period (434 dpt), 43.6% of the initially lordotic fish presented a completely normal external phenotype (Fig. 6A). At the same time, in 9.3% of the cases, the initially abnormal morphology was turned to uncertain (Fig. 6A). A similar recovery trend was observed in the case of fish with an uncertain external phenotype at 1 dpt, where a substantially higher recovery rate was detected (67.6% at 77dpt, 73.5% at 434 dpt, Fig. 6B).

**Figure 7:**
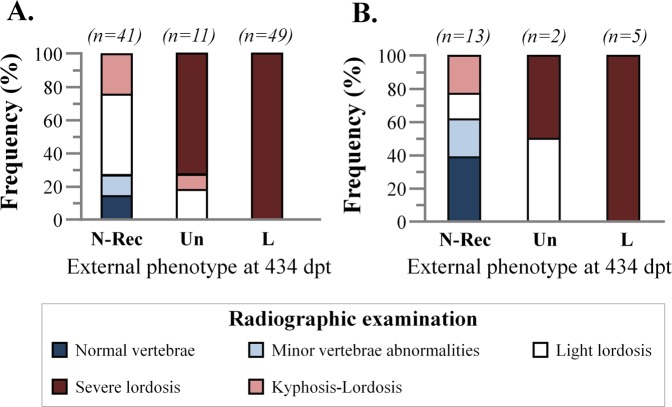
Radiographic categorization of the fish at the end of the on-growing period (434 days post-tagging). (**A**) Fish with lordotic external morphology at 1 dpt. (**B**) Fish with uncertain external morphology at 1 dpt. N-Rec, fish with a normal external morphology. Un, fish with uncertain external morphology. L, fish with lordotic external morphology. Representative images of the different radiographic phenotypes are given in Fig. 8.

**Figure 8:**
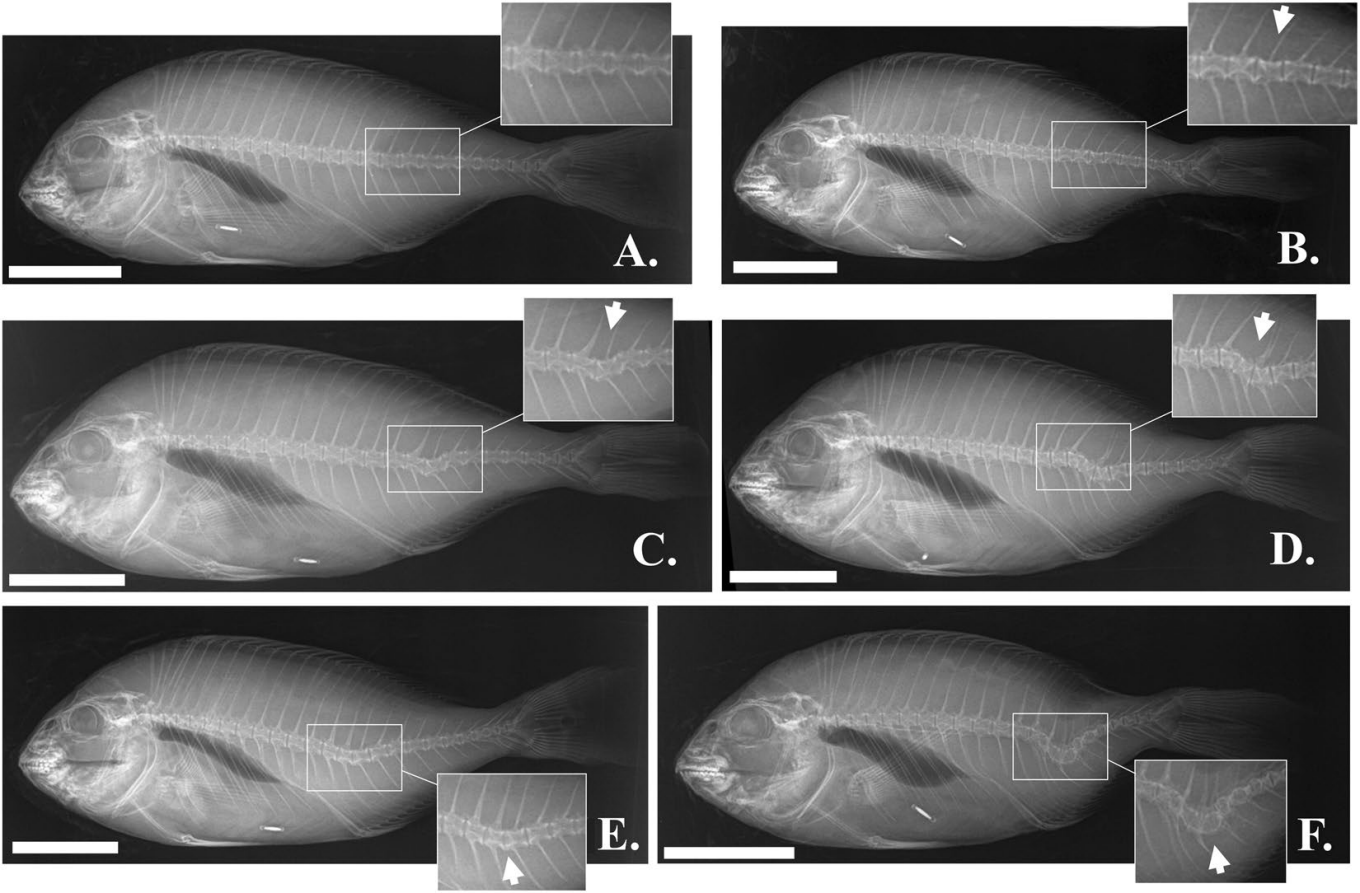
Haemal lordosis (C–F) of variant severity in seabream at the end of the on-growing period (434 days post-tagging). (**A**) Normal fish. (**B**) Normal fish with minor abnormalities of individual centra (arrow). (**C**) Fish with light internal lordosis and a normal external phenotype. (**D**) Fish with a kyphosis anterior to lordosis. (**E**) Fish with an uncertain external morphology and a severe internal lordosis. (**F**) Fish with an abnormal external morphology and a severe internal lordosis. Scale bars are equal to 5 cm.

Recovery of haemal lordosis was verified by the following radiographic examination of the fish at the end of the experiment (434 days post-tagging, dpt). Among fish with recovered external morphology, 26.8% presented a complete recovery of the vertebral column, without any abnormality of individual vertebrae (14.6%, Figs 7A and 8A), or with minor abnormalities of individual centra (12.2%, Figs 7A and 8B). The 48.8% of the fish with recovered external morphology presented a light lordosis (Figs 7A and 8C), whereas 24.4% of them showed a counterbalancing kyphosis anteriorly to lordosis (Figs 7A and 8D). The majority (72.7%) of lordotic specimens which turned to uncertain external appearance at 434 dpt, presented a severe lordosis (Figs 7A and 8E). The partial recovery of haemal lordosis, was also radiographically verified in the case of fish with initially uncertain phenotype (Fig. 7B). Finally, severe vertebral lordosis was present on the radiographies of all the specimens with lordotic external morphology at 434 dpt (Figs 7A and 8F).

### Effect of lordosis on the body shape of seabream at the beginning and the end of on-growing period

**Figure 9:**
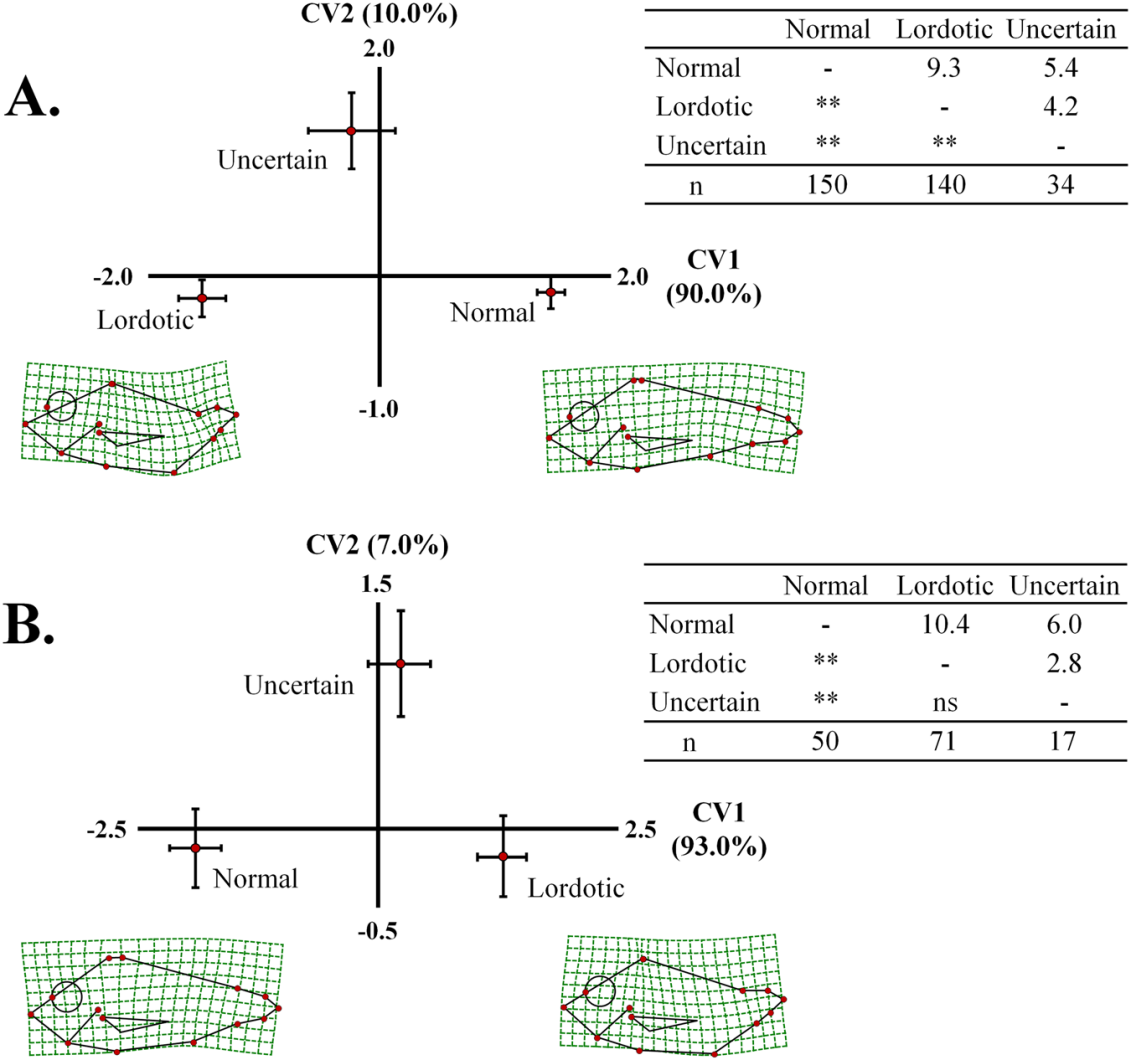
Distribution of the fish with normal, lordotic or uncertain external morphology along the two axes of canonical variate analyses (CV1, CV2). (**A**) Beginning of the on-growing period (1 day post-tagging). (**B**) End of the on-growing period (434 days post-tagging). Means (±2SE) of the canonical scores are given. Numbers in brackets are equal to the percentage of shape variance explained by each canonical axis. Spline diagrams demonstrate the components of shape change relative to the extreme values (X1) of CV1. Squared Mahalanobis distances between the different groups and the respective significance levels are given in the tables next to each analysis. ns, p > 0.05. **p < 0.01. n, number of specimens in each group.

Canonical variate analysis revealed that lordosis significantly affected the body shape of seabream at both 1 and 434 days post-tagging (Wilks λ = 0.262 and 0.261 respectively, p < 0.001, Fig. 9). In both analyses, the first canonical axis (CV1) explained the majority of phenotypic variance (90.0–93.0%), discriminating normal from lordotic fish. Fish of uncertain phenotype were distributed between normal and lordotic groups (Fig. 9). In the first sampling period, squared Mahalanobis distances were significantly different between all the examined groups (Fig. 9A), whereas in the final sampling only normal fish were significantly different from the lordotic and uncertain groups (Fig. 9B). Spline diagrams demonstrated that abnormal fish were characterized by a ventral transposition of the anterior base of the anal fin (landmark 7) and by a dorsal-anterior shift of the caudal peduncle (landmarks 2–6, Fig. 9).

### Morphometric analysis of the recovery of haemal lordosis

**Figure 10:**
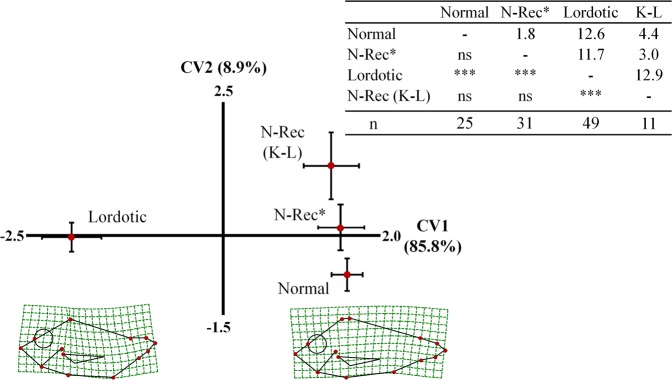
Distribution of the fish of the fifth sample (434 days post-tagging) with a recovered normal (N-Rec*, N-Rec K-L), normal since the 1 dpt (Normal), or a lordotic (Lordotic) phenotype along the two axes of canonical variate analysis (CV1, CV2). Means (±2SE) of the canonical scores are given. Numbers in brackets are equal to the percentage of shape variance explained by each canonical axis. Spline diagrams demonstrate the components of shape change relative to the extreme values (X1) of CV1. Squared Mahalanobis distances between the different groups and the respective significance levels are given in the table next to the graph. ns, p > 0.05. ***p < 0.001. N-Rec*, N-Rec fish of Fig. 6A, excluding the K-L. N-Rec (K,L), K,L fish of Fig. 7A. Lordotic group consisted of the fish of Fig. 6A with severe lordosis. n, number of specimens in each group.

Following radiographic examination of fish at the end of the on-growing period (Fig. 7A), we examined whether fish with a recovered normal phenotype differ in body shape from those that were normal since the beginning of the on-growing period (1 day post-tagging, dpt). First axis (CV1) of the canonical variate analysis explained 85.8% of the total variance and discriminated fish with a normal from those with a lordotic phenotype (Wilks λ = 0.155, p < 0.001, Fig. 10). Squared Mahalanobis distances were significant only between lordotic and normal fish (Fig. 10). Distance between fish with a recovered normal phenotype and fish that were normal since the beginning of the on-growing period (1 dpt), was not significant (Fig. 10).

**Figure 11:**
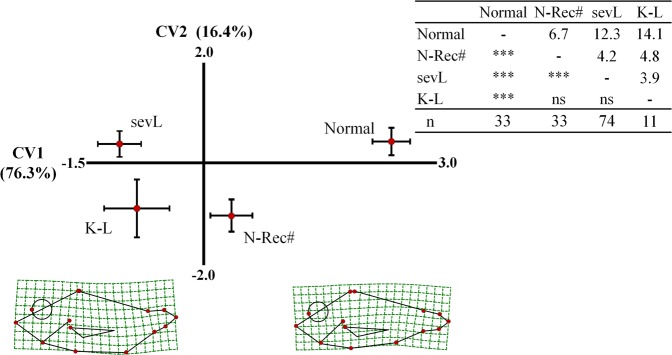
Distribution of lordotic juveniles at the beginning of on-growing (1 day post-tagging) along the first two axes of canonical variate analysis (CV1, CV2). According to their external morphology and radiographic appearance at the end of on-growing period (434 days post-tagging, Fig. 6A), juveniles were categorized into fish with severe lordosis (sevL, Fig. 7A), fish with a recovered normal external phenotype and an internal kyphosis-lordosis (K,L, Fig. 6A), fish with a recovered normal external morphology (N-Rec#, N-Rec and Un fish of Fig. 6A, excluding the K,L and “severe lordosis”), and normal fish since the beginning of the on-growing period (Normal). Means (±2SE) of the canonical scores are given. Numbers in brackets are equal to the percentage of shape variance explained by each canonical axis. Spline diagrams demonstrate the components of shape change relative to the extreme values (X1) of CV1. Squared Mahalanobis distances between the different groups and the respective significance levels are given in the table next to the graph. ns, p > 0.05. ***p < 0.001. The sevL group consisted of the 57 fish of Fig. 6A (“Severe Lordosis”) and another 17 fish with lordotic external morphology, which were not x-rayed. n, number of specimens in each group.

Lordotic juveniles (1 day post-tagging, dpt) were classified according to their morphological evolution into juveniles that maintained the external lordotic phenotype (sevL) up to the end of the on-growing period (434 dpt), and juveniles that presented a recovered normal phenotype at 434 dpt (N-Rec#, Fig. 11). First axis (CV1) of the canonical variate analysis explained 76.3% of the total variance and discriminated fish with a normal phenotype from lordotic sevL and N-Rec# groups (Wilks λ = 0. 420, p < 0.001, Fig. 11). Squared Mahalanobis distances were significant between normal and lordotic fish, independently of the morphological evolution of the latter (Fig. 11). Interestingly, significant body shape differences were detected between lordotic sevL and N-Rec# juveniles (squared Mahalanobis distances, Fig. 11). Spline diagrams demonstrated that shape differences between sevL and N-Rec# lordotic juveniles mainly concerned the severity of the deviation from normal fish (Fig. 11). Use of functions from the canonical variate analysis for the classification of individuals resulted to a successful reclassification of the 94%, 89% and 73% of normal, sevL and N-Rec# fish respectively (Table 3).

**Table 3:** Classification of the juveniles of Fig. 11 to the different phenotypic categories, based on their morphometry and the classification functions of the canonical variate analysis.

	sevL	N-Rec#	Normal	K-L	Total
sevL	**89**	7	3	1	100
N-Rec#	21	**73**	6	0	100
Normal	0	6	**94**	0	100
K-L	45	9	0	**46**	100

A priori classification probabilities were proportional to the initial group sizes. Numbers indicate percentages of correct reclassification (bold) or mis-reclassification of the specimens.

## Discussion

This study shows that external morphology of lordotic seabream recovers during the on-growing period. The observed recovery rate depended on the initial abnormality severity and ranged from 43.6% in case of fish with severe lordosis, to 73.5% in case of fish with light severity of lordosis. Interestingly, radiographic examination of fish showed that the recovery of lordotic external morphology was accompanied by a partial to complete recovery of the vertebral column. To our knowledge this is the first study documenting the recovery of severe axis abnormalities in fish. In a similar study, Witten *et al*.^31^ demonstrated a remodeling of fused vertebral centra into one non-deformed centrum, which however continued presenting multiple haemal and neural processes.

Decrease of abnormalities prevalence during fish growth is not rare. It is attributed to an increased mortality rate of the abnormal individuals^3,10,15,32^, or in case of gill-cover abnormalities of light intensity, to the recovery of abnormal phenotype^16,17^. In the present study, the decreased incidence of lordosis during the growth of Gilthead seabream can not be attributed to a lethal effect of the deformity, since differences in the mortality rate between normal and lordotic fish were not significant. Moreover, the morphological monitoring of pit-tagged individuals during on-growing period revealed that the decrease in the incidence of abnormality was a result of recovery of the lordotic phenotype. In support of this conclusion, at the end of the on-growing period, geometric morphometric analysis showed that there were no significant differences in body shape between fish with a recovered phenotype and those with a normal phenotype since the beginning of the study (Fig. 10). Finally, the recovery of lordosis was also supported by the radiographic examination of fish at the end of the on-growing period (Fig. 7).

In species which are marketed as a whole, establishment of a link between external phenotype and skeleton is a significant goal for the quality assessment of reared fish, since consumers decisions are made on the external morphology^26,33^. Alterations of body-shape by the presence of haemal lordosis have a continuous range, depending on the angle of the vertebral column at the affected area, and by the number of abnormal vertebrae^18,23^. With respect to the external morphology, haemal lordosis presents a clear phenotype with a shorter and dorsally shifted caudal penducle, as well as a ventral shift of the posterior abdominal area^18,34^. In the present study, dorsal shift of the caudal peduncle was used to discriminate lordotic from normal individuals. This method of morphological categorization was successfully validated by radiographic analysis of the juvenile sample taken at the beginning of the study, with 100% of the fish with a lordotic external morphology presenting lordosis of the vertebral column. Applied methodology only failed to correctly categorize 5% (4 out of 78) of the fish with a normal external morphology and a lordotic vertebral column of very light severity (Fig. 3). Significant differences in body shape between juveniles with a normal, lordotic or uncertain external phenotype furthermore supported the initial categorization of the fish (Fig. 9).

Finfish skeleton is subject to continuous resorption, remodeling and reshaping. These processes are essential for development, growth, repair and adaptation of skeleton to mechanical loads^35^. Haemal lordosis is known to result from the excess mechanical loads of muscles on the vertebral column, during the swimming of the juveniles in hatchery tanks with relatively high water-current speed^21–23^. Since lordotic vertebrae are characterized by increased bone volume, flattened dorsal zygapophyses and extra lateral ridges, lordosis has been suggested as an adaptive response of the vertebral column to the new regime of increased loads^36^. In the present study, lordosis recovery is shown to be the consequence of two different processes; partial to complete repair of the vertebral column (in 75.6% of the fish with a recovered external morphology), and development of counteracting kyphosis-like bending of the vertebral column (Fig. 8D, in the 24.4% of the fish with a recovered external morphology). Both processes might be triggered by fish transfer to sea cages, an environment of -comparatively to the tanks- less intense water current velocities. Under this hypothesis, observed lordosis-recovery could be a result of the adaptation of growing vertebrae to the new swimming environment.

Despite shape alterations of the deformed vertebrae, histopathological organization of lordotic vertebral centra involves the presence of a fibrous cartilage replacing cancellous and compact bone, enlargement of trabecular spaces and a reduction of the notochordal lumen^37^. Histological processes that took place during the recovery of lordotic vertebrae in the present paper remain unknown. In the only known similar study on the recovery of vertebral fusion in Atlantic salmon, recovered vertebral bodies acquired the typical normal radiographic appearance, following an histological process involving remodeling of notochordal and cartilage tissues and reshaping of the fused vertebral centra^31^.

In marine finfish aquaculture, phenotypic quality of the juveniles at the end of the hatchery phase (1–10 g mean weight) has been widely suggested and used as a precise predictor of the phenotypic quality at the end of the on-growing period (>300 g mean weight)^1,34,38^. Our results clearly suggest that quality control at the end of the hatchery phase has to take into account the recovery potential of haemal lordosis. Interestingly, as it was shown by body shape analysis (Fig. 11), identification of lordotic juveniles which are expected to recover during on-growing is possible through geometric morphometrics. In future, the automatic sort-out of lordotic juveniles with low recovery potential could be possible through the incorporation of computer-assisted systems in the quality control of reared fish.

## Supplementary information

Supplementary Information

Dataset 1

## Data Availability

All data generated and analysed during this study are included in this published article (and its Supplementary Information files).
