# Supplementary material for: Recovery of haemal lordosis in Gilthead seabream (Sparus aurata L.)
Source: Sci Rep. 2019 Jul 8;9:9832. doi: 10.1038/s41598-019-46334-1 (PMC6614392; doi:10.1038/s41598-019-46334-1)
Supplement: Supplementary file 1 — Supplementary Information [file 41598_2019_46334_MOESM1_ESM.doc]

**Recovery of haemal lordosis in Gilthead seabream (*Sparus aurata* L.)**

Stefanos Fragkoulis^1^, Alice Printzi^1^, George Geladakis^1^, Nikos Katribouzas^2^, George Koumoundouros^1*^

**1** Biology Department, University of Crete, Herakleion, Crete, Greece

**2** Andromeda S.A., PEO Patron-Athinon 55, Agios Vasilios, 26500 Rion, Greece

*, corresponding author, [gkoumound@uoc.gr](mailto:gkoumound@uoc.gr)


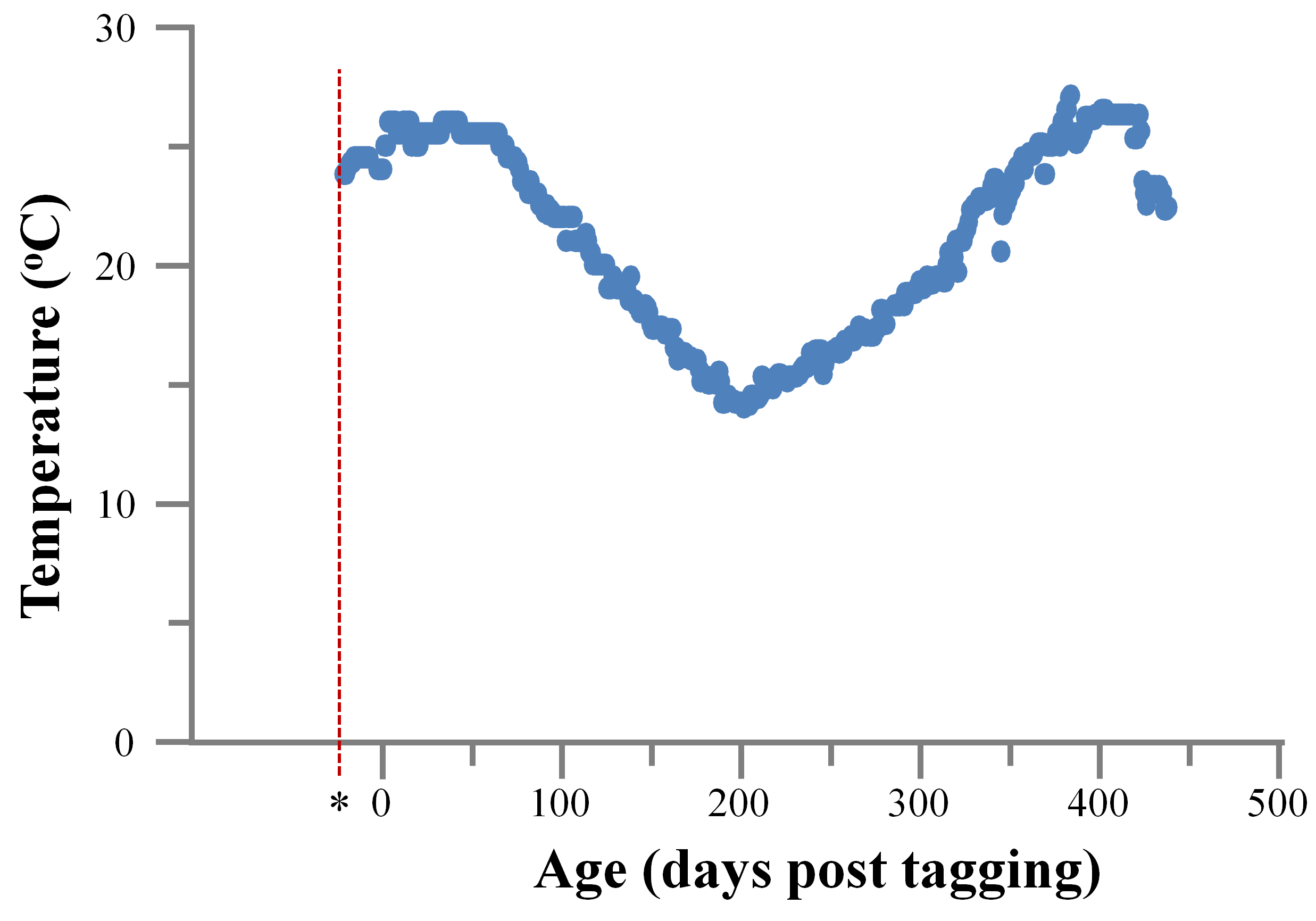


**Figure S1.** Fluctuation of water temperature during the on-growing period. *, fish transfer in the sea cages.
